# 3D Quantitative Imaging of Unprocessed Live Tissue Reveals Epithelial Defense against Bacterial Adhesion and Subsequent Traversal Requires MyD88

**DOI:** 10.1371/journal.pone.0024008

**Published:** 2011-08-25

**Authors:** Connie Tam, Jeffrey LeDue, James J. Mun, Paul Herzmark, Ellen A. Robey, David J. Evans, Suzanne M. J. Fleiszig

**Affiliations:** 1 School of Optometry, University of California, Berkeley, California, United States of America; 2 Program in Vision Science, University of California, Berkeley, California, United States of America; 3 Department of Molecular and Cell Biology, University of California, Berkeley, California, United States of America; 4 College of Pharmacy, Touro University California, Vallejo, California, United States of America; 5 Programs in Infectious Diseases and Immunity and Microbiology, University of California, Berkeley, California, United States of America; South Texas Veterans Health Care System, United States of America

## Abstract

While a plethora of *in vivo* models exist for studying infectious disease and its resolution, few enable factors involved in the maintenance of health to be studied *in situ*. This is due in part to a paucity of tools for studying subtleties of bacterial-host interactions at a cellular level within live organs or tissues, requiring investigators to rely on overt outcomes (e.g. pathology) in their research. Here, a suite of imaging technologies were combined to enable 3D and temporal subcellular localization and quantification of bacterial distribution within the murine cornea without the need for tissue processing or dissection. These methods were then used to demonstrate the importance of MyD88, a central adaptor protein for Toll-Like Receptor (TLR) mediated signaling, in protecting a multilayered epithelium against both adhesion and traversal by the opportunistic bacterial pathogen *Pseudomonas aeruginosa ex vivo* and *in vivo*.

## Introduction

Many of our tissue surfaces are lined with epithelia that are thought to be a first line defense against infection. Yet the mechanims involved in epithelial barrier function against microbial traversal *in vivo* remain poorly understood, in part due to the paucity of suitable *in vivo* models. Attempts to understand factors that modulate microbe traversal of epithelia have been limited to *in vitro* experiments, most utilizing monolayers of cultured cells grown on permeable filters, and with an emphasis on establishing contributing virulence factors rather than determining epithelial defenses against it [1]–[3]. While it is known that epithelial cells do express factors with the potential to be defensive [4], [5], those that actually participate in limiting bacterial trafficking through cell multilayers are yet to be determined. Moreover, relevance of *in vitro* studies using epithelial cells grown in culture to actual *in vivo* barrier function of the epithelium is not yet certain.

There have been two major obstacles to the development of *in vivo* models to study tissue traversal by microbes and the defenses that protect against it. One is that “infection” models used by most investigators are disease models in which early interactions between bacteria and the epithelium are deliberately by-passed to induce disease. Understanding factors that maintain health at the surface of epithelial-lined tissues, such as those that defend against microbial traversal, require different models, and the use of outcome measures more subtle than scoring morbidity/mortality or quantification of total microbe load within the tissue. Related to this, the other major obstacle has been a lack of techniques for accurately localizing bacteria within live *in vivo* infected tissue. Fixation and sectioning of tissue to view microbe penetration in cross-section enables viewing of only a small area, introducing the possibility that non-representative areas are studied, or conversely, that rare but important features are missed. Other disadvantages of fixation are that it removes the opportunity to gain temporal information, and that it can impact morphology or introduce artifacts such that microbial location may be misinterpreted.

The healthy corneal epithelium at the surface of the eye usually resists bacterial adhesion, which is thought to involve the presence of surface-associated mucins [6], [7]. Recently we developed a means to enable bacteria to adhere to the epithelium of mouse corneas that also allowed the epithelium's resistance to subsequent traversal by the adherent bacteria to be manipulated. This involved blotting the epithelial surface with a lint-free tissue prior to bacterial inoculation to enable bacterial adhesion. EGTA (Ethylene Glycol Tetraacetic Acid) treatment after this blotting process can then be used to toggle on epithelial susceptibility to traversal by adhering bacteria. Since EGTA is removed (by washing) prior to bacterial inoculation, the impact of this chemical treatment on traversal is likely to involve its effects on the corneal epithelium rather than on the bacteria. EGTA, a calcium-chelator, is capable of disrupting calcium dependent cell-cell junctions [8]. However, calcium is also required for many other cell functions, including various innate defense responses [9]. Thus, the mechanisms by which EGTA promotes susceptibility to epithelial traversal by adherent bacteria is yet to be determined.

Toll-like receptors (TLRs) are a class of receptors that bind microbial ligands (e.g. Microbial-Associated Molecular Patterns or MAMPS) or damage-associated molecular patterns (DAMPS) to trigger innate immune responses in multiple cell types including epithelia [10]–[12]. While it is known that some factors made in response to TLR-mediated signaling have direct antimicrobial activities (e.g. defensins) [5], [13], [14], the role of these, or other factors, in limiting microbial traversal through tissue epithelia has not been directly explored using actual tissue models.

Here, we applied a suite of methods that together enable the position of bacteria within freshly excised, unfixed and unprocessed tissue to be readily visualized and quantified in 3D, and as a function of time. Using these methods, we found that MyD88, a central adaptor protein for TLR and IL-1R-dependent signaling critical in pathogen recognition, and subsequent innate immune defense responses [15], is required for epithelial resistance to both adhesion and traversal by the Gram-negative bacterium *P. aeruginosa*. These data suggest that epithelial defense against colonization *during health* involves innate defense responses that are known to be regulated. Further, the methods and results outlined in this report advance the range of tools available for studying microbial traversal of epithelia and the host factors that normally protect against it.

## Results and Discussion

### Localization of bacteria within the epithelium of fresh whole tissue

The tissue we chose to use for this study, the corneal epithelium, is a multilayer of epithelial cells that covers the cornea of the eye. The cornea lends itself to imaging because it is both external and transparent. Epithelial cells on the corneal surface are viable (non-keratinized), and are normally bathed in fluid (tears) allowing the use of a water immersion objective with minimal disruption to normal physiology.

The healthy corneal surface does not normally bind bacteria or other microbes. Studying bacterial interactions with the corneal epithelium thus necessitates some form of manipulation to enable bacteria to adhere. As discussed above, superficial blotting with tissue paper can be used for that purpose, and it is minimally disruptive to tissue architecture [16]. Subsequent epithelial traversal by the adherent bacteria can then be toggled on (or off), by use (or omission) of EGTA treatment after the blotting procedure.

**Figure 1:**
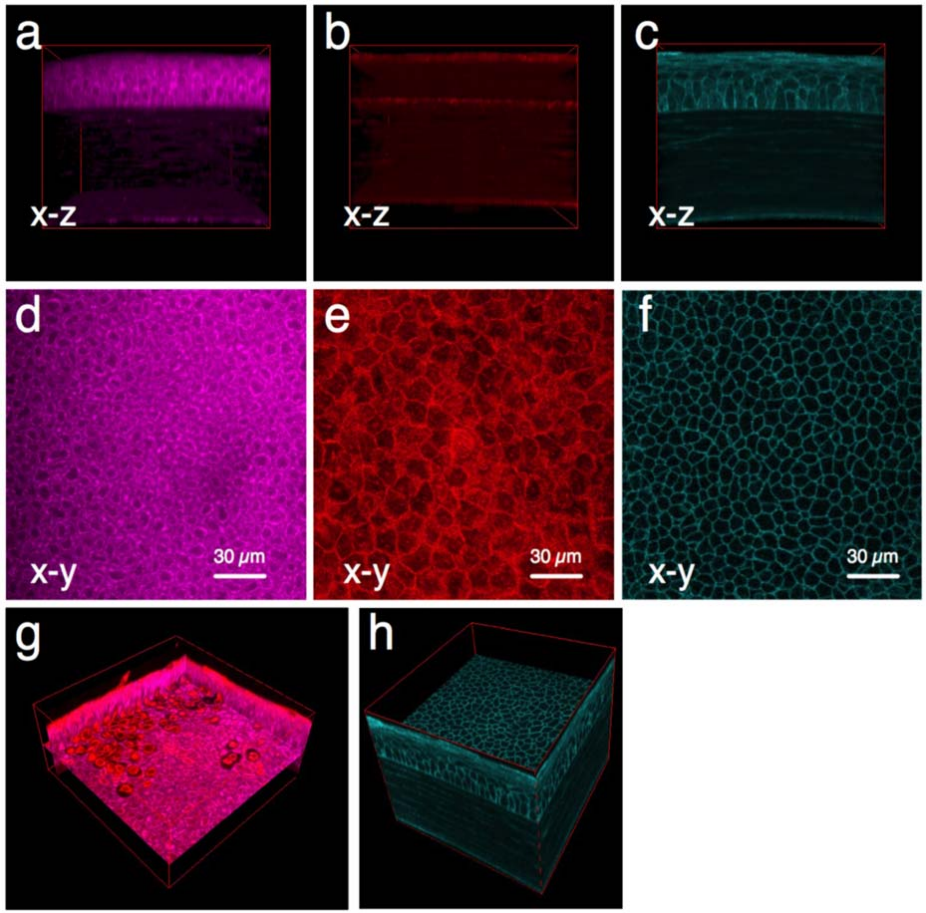
3D imaging of mouse corneas using freshly excised mouse eyeballs. Three different techniques were used to visualize cells in all layers of the cornea. Panels (**a**) and (**d**) show two-photon excitation of cytoplasmic NAD(P)H to reveal metabolically active (live) cells in the epithelium and endothelium of the cornea by autofluorescence (magenta). Panels (**b**) and (**e**) show reflection confocal imaging (red) for localization of epithelial and endothelial cells (including live and dead cells), and collagen fibers in the stromal region (red). Panels (**c**) and (**f**) show confocal imaging of CFP-tagged cell membranes (cyan) expressed in transgenic mice. All three imaging methods enabled visualization of distinct cell layers in intact corneas of at least 150 µm thickness (i.e. mouse). Panel (**g**) shows two-photon autofluorescence and reflection images overlaid to distinguish live and dead cells. Panel (**h**) shows that 3D images of CFP-membranes exhibit a high signal-to-background ratio providing excellent contrast for bacterial localization experiments. Field (xy) size: 189 µm x 189 µm.

With methods in hand to encourage bacteria to interact with the epithelium, the next goal was to develop methods for visualizing/localizing bacteria within the bacterially-challenged tissue that do not require processing (i.e. fixation, labeling, staining) of the tissue. Details of the methods utilized are outlined in the materials and methods. Briefly, bacteria were visualized using a multicopy plasmid expressing enhanced green fluorescent protein (GFP). Corneal epithelial cells within bacterially challenged, and then freshly excised unprocessed eyeballs, were imaged using one or more of three different techniques: 1) NAD(P)H autofluroescence (AF) (multiphoton, images cytoplasm of metabolically active cells). 2) A reflection technique (confocal, images both live and dead cells). 3) Use of transgenic mice expressing membrane-bound cyan fluorescent protein (CFP) (confocal, enables cell membranes to be visualized). All three methods enabled cells within all layers of the cornea (epithelium, stroma and endothelium) to be visualized (Figures 1A, B, C). Thus, it was possible to accurately localize the upper and lower limits of each layer, providing landmarks for quantifying the depth of microbe penetration using any of the three methods.

Each of the three imaging modalities had unique features for gaining more detailed information. For example, since NAD(P)H resides within the cytoplasm, the NAD(P)H AF method enables subcellular imaging; the cytoplasm fluoresces, while the nucleus and plasma membrane appear black in contrast (Figure 1D). Since its fluorescence intensity is proportional to cellular metabolic activity [17]–[19], this technique also provides information about the health of individual cells (Figure 1D). The reflection confocal and membrane-CFP methods each allow positive (rather than negative) visualization of epithelial cell boundaries, and thus can be used to ensure that both dead and live cells are accounted for (Figures 1E, F). Thus, when used in combination with NAD(P)H AF, confocal or membrane-CFP can enable live and dead cells to be distinguished from one another (e.g. Figure 1G). The AF and reflection methods (in contrast to membrane CFP) do not require a specific type of genetically modified animal, which means that any animal can be studied, including wild-type and all genetically modified species. Membrane-bound CFP, on the other hand, provides a high signal-to-background ratio, and consequently a high image contrast of cell membranes (Figures 1F, 1H and Video S1), compared to either AF or reflection (Figures 1D, E respectively), making this an ideal method for determining if infecting bacteria are intracellular or extracellular.

**Figure 2:**
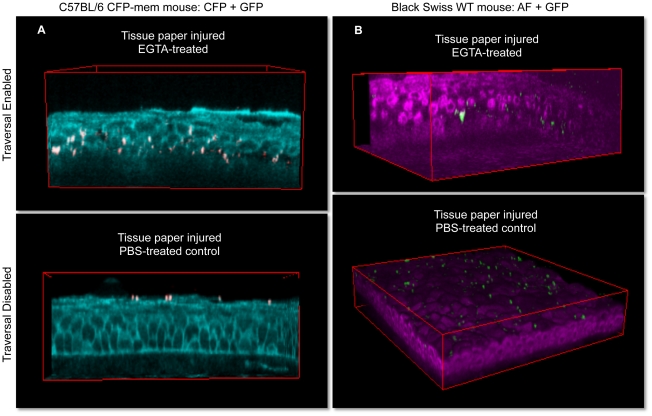
3D imaging of bacterially challenged eyeballs. All eyeballs were tissue paper blotted to enable bacterial adherence to the corneal epithelium. Susceptibility to traversal by adherent bacteria was then toggled on/off by use/omission of EGTA treatment following the blotting procedure. Upper images (EGTA used to toggle on traversal) show deep bacterial traversal 6 h after bacterial challenge in EGTA-treated corneas. Lower images were PBS-treated controls (EGTA step omitted) and showed bacterial adherence to the surface without subsequent bacterial traversal. Panel (**a**) shows confocal technique using mice with CFP-tagged cell membranes (cyan), and panel (**b**) shows two-photon images of cellular NAD(P)H autofluorescence (magenta) in transgenic (C57BL/6 background) or wild-type (Black Swiss background) mouse corneas respectively. Field size: 189 µm × 189 µm.

Images from bacterially-challenged corneas (6 h) are shown in Figure 2. The top panels show results for corneas that were first blotted and then treated with EGTA for 1 h prior to bacterial challenge. Both the confocal CFP membrane method (Figure 2A and Video S2) and the NAD(P)H autoflurescence method (Figure 2B and Video S3) revealed epithelial traveral by the bacteria; i.e. many bacteria were detected deep within the multilayered corneal epithelium. Consistent with our previously published findings showing that the the basal lamina between the epithelium and the stroma functions as a barrier to bacterial passage [20], few bacteria were noted in the underlying stroma. Also consistant with our previous results [16], corneas that were not subsequently treated with EGTA prior to bacterial challenge (blotted only, bottom panels and Video S4) were susceptible to bacterial adhesion only, without bacterial penetration beyond the epithelial surface.

**Figure 3:**
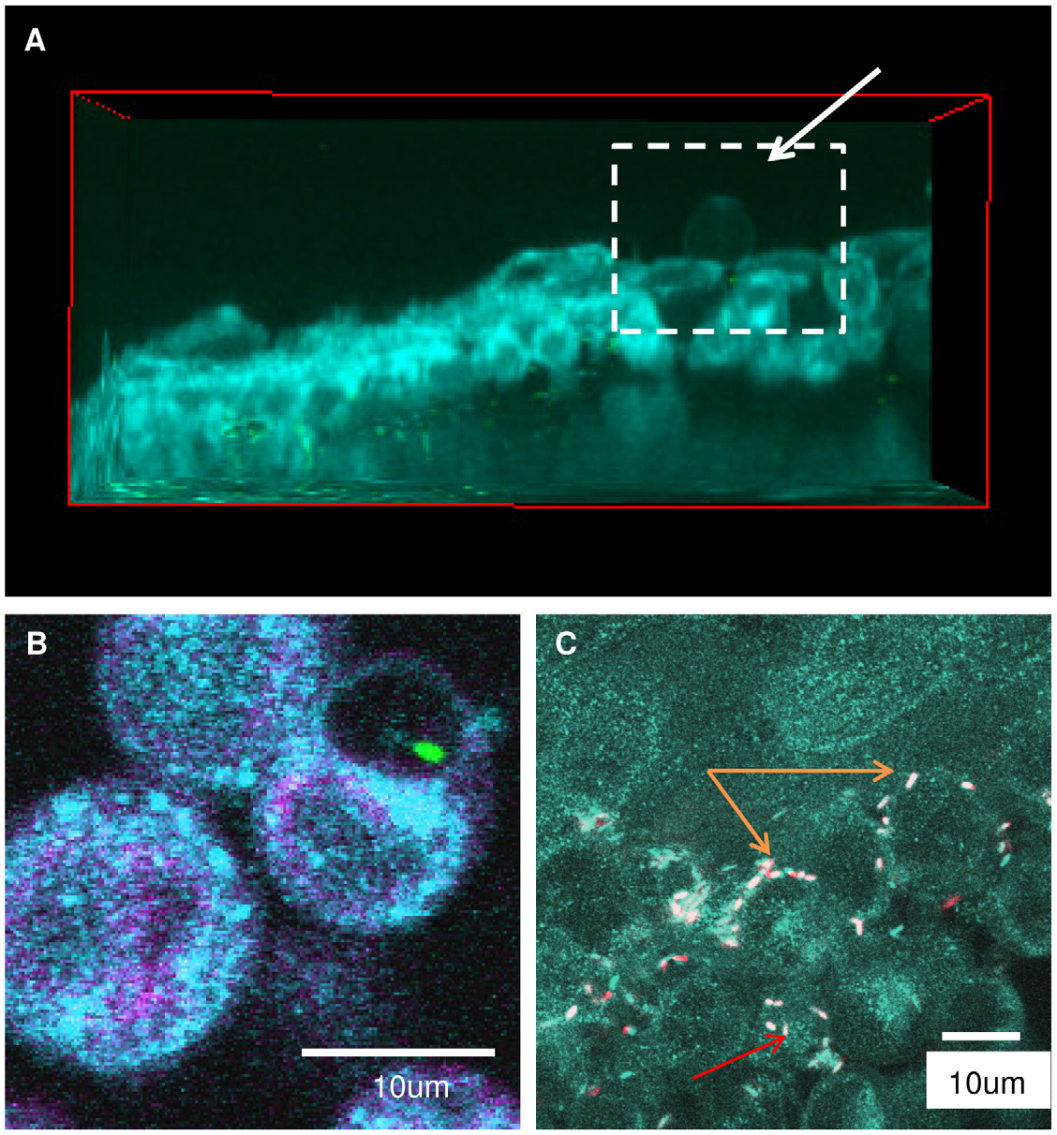
Temporal and spatial tracking of EGTA-enabled bacterial traversal. Traversal was enabled in the corneal epithelium of transgenic mice expressing CFP-tagged cell membranes (cyan). Panels (**a, b**) show bacterial-induced membrane bleb formation *ex vivo* visualized as spherical membrane projections (arrow) extending away from the epithelial cells. A representative view in the xz plane is shown in (**a**), and a higher magnification imaging revealing a bleb-confined bacterium is shown in (**b**). In (**c**), bacteria can be seen located between cells (orange arrows) where some were motile (fast-moving GFP-bacteria, red; slower-moving GFP-bacteria, white  =  captured twice in both CFP and GFP channels). Other bacteria in this image appeared to be in the cytoplasm (red arrow).

Having established methods for enabling and also visualizing bacteria deep within unprocessed and unsectioned live tissue, we next explored if these methods could be used to provide more detailed spatial and temporal information about the traversal process and its impact. Use of the CFP membrane mouse and high magnification enabled the position of bacteria relative to cell membranes, and the status of those membranes, to be determined. For example, perusal of images in Figures 2 reveals that the CFP signal is relatively diffuse after bacterial traversal of blotted/EGTA-treated corneas relative to the very clearly localized CFP signal seen in the uninfected eye in Figure 1. Blotting/EGTA treatment used alone without bacterial challenge did not cause CFP diffusion (data not shown), suggesting that it was the bacterial traversal *per se* that impacted the appearance of the cell membrane marker; perhaps not surprising considering that *P. aeruginosa* can elaborate multiple toxins [21] and basolateral cell surfaces are highly susceptible to them [22]. Use of the CFP-membrane mouse also revealed that *P. aeruginosa* can induce the formation of membrane blebs, and can localize within them, a phenomenon which we have reported to occur when *P. aeruginosa* infects cultured corneal epithelial cells [23], [24]. Figures 3A and 3B each show examples of membrane blebs; the higher magnification used in Figure 3B enabled a bacterium to be located within a bleb. While the significance of “bleb niche” formation in epithelial cells during *P. aeruginosa* infection is still under investigation, this finding that they occur in actual infected fresh tissue, and not only in cells grown and infected in tissue culture wells, lends justification to that effort.

One clear advantage of imaging live versus fixed tissues is that temporal information can be obtained using the same sample. For example, individual bacteria and cells within the epithelium of fresh tissue can be tracked over time for their location and/or their viability (Figures 3B, 3C and Video S5). Video S5 shows that the bacterium located within the membrane bleb in Figure 3B is exhibiting swimming motility inside the bleb, confirming another phenomenon that we have reported with cultured cells, but this time for a cell located within the corneal epithelium of a live eyeball. Figure 3C (high magnification z -stack) shows several bacteria in the basal (deep) layers of the corneal epithelium of intact eyeballs mostly located *between* epithelial cells. In this image moving bacteria are shown in red, while white bacteria are stationary since they were captured twice (sequentially in CFP and GFP channels) in the same z-plane (temporally spaced images were overlaid). In addition to providing insights into the temporal details of bacteria-cell interactions, these methods could also be used to monitor individual host cell responses to bacterial challenge over time (e.g. by morphology or by tracking NAD(P)H AF), and with respect to the position of individual bacteria.

**Figure 4:**
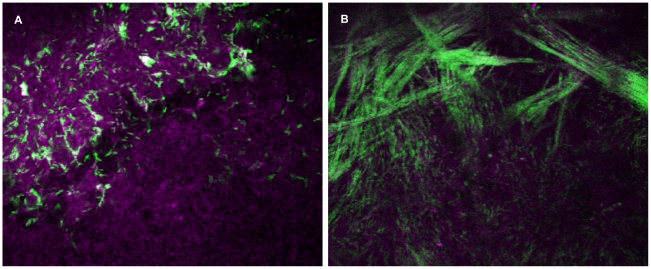
Imaging of bacteria and cells within opaque infected mouse corneas. Panel (**a**) shows NAD(P)H autofluorescence (magenta) of corneal epithelial cells and bacteria (green) detected from optically opaque (infected) mouse eyes 24 h post-scratch and inoculation. In panel (**b**), bacteria (green) within the stroma of the cornea were found to orient themselves along the pattern of the collagen fibrils. Field size: 189 µm × 189 µm.

Lack of significant bacterial penetration into the stroma as a result of an intact basal lamina likely explains why blotting/EGTA treatment is not sufficient to enable susceptibility to overt (clinically visible) disease despite bacterial traversal through the epithelium [16]. We next explored the usefulness of these imaging methods when there is overt disease, i.e. when the cornea becomes optically “opaque” (non-transparent) due to the infiltration of leukocytes, e.g. neutrophils and monocytes [25]–[27]. Disease was induced by scratching the cornea through to the level of the anterior stroma (damages basal lamina barrier) prior to challenging with bacteria [28], [29]. It remained possible to detect cellular NAD(P)H AF and to localize GFP-labeled *P. aeruginosa,* even when the infecting bacteria were located deep within the stromal region of these opaque corneas (Figure 4, Video S6). The images collected revealed interesting insights illustrating the power of this technology compared to fixed/sectioned tissue. For instance, the images shown in Figures 4A, 4B and Video S6 were collected at a region of the cornea where the basal lamina was not directly impacted by the scratching process. This z-stack was collected with the optical axis of the microscope parallel to, but laterally shifted from, the optical axis of the eye and as a result of the curvature of the eye, the image captured in Figure 4A (taken from the stack) shows the epithelium at the top and the interface between the epithelium and the stroma (basal lamina region) towards the bottom. This enabled direct visualization of the “filtering” effect of the basal lamina, i.e. bacteria can be seen deep in the epithelium arranged in circular patterns surrounding cells/nuclei with concentrations tapering off at, and under, the basal lamina. While the images collected directly under the basal lamina showed a distinct bacteria free zone, deeper regions of the stroma again revealed the presence of bacteria, this time arranged in a different pattern. Here, bacteria were found radiating out laterally in single file, end-to-end lines, corresponding with orientation of the stromal collagen fibrils (Figure 4B, Video S6). Images collected from adjacent regions showed that these bacterial “trains” originated at the scratched region where bacteria enter the corneal stroma directly through the damaged basal lamina, and then eminated away from the area in multiple directions. Whether *P. aeruginosa* utilizes a form of surface-associated motility along collagen fibrils to disseminate through the cornea is to be determined. This is of interest, considering that twitching motility is a critical virulence factor for *P. aeruginosa* in this corneal infection model [30], [31].

### Impact of MyD88 on bacterial traversal

To gain further mechanstic insights into how the corneal epithelium normally defends itself against bacterial traversal, the role of MyD88, a central adaptor protein for TLR signaling, was examined. This was done by comparing wild-type to MyD88 knockout (−/−) mice.

**Figure 5:**
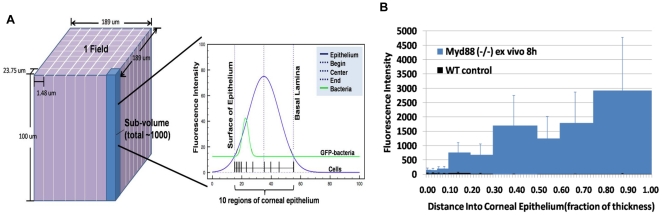
Quantitative localization of bacteria within *ex vivo* blotted and bacterially challenged MyD88 deficient mouse corneas at 8 h time-point. Panel (**a**) shows a schematic of the custom bacterial quantification program. Each image stack is divided into ∼1000 sub-volumes; in each sub-volume, the top and bottom of epithelium were localized in which 10 bins along the z-depth were assigned, followed by bacterial fluorescence quantification in each bin, and finally summation of all bins of the same z-depth across the entire image stack. Panel (**b**) shows quantitative analysis of bacteria traversing to the basal lamina for MyD88 (−/−) but not wild-type mice (0.00 represents the corneal surface, 1.00 the basal lamina). Average ± S.E. of three fields are shown.

After image acquisition (z-stacks), image stacks were analysed using a custom program which permited quantitative z-axis profiling of bacterial traversal from any of the three signals that localized the structure of epithelium in addition to bacterial fluorescence (Figure 5A). To take tissue surface irregularities into account (the corneal surface is curved), an image stack (a field) was divided into approximately 1000 sub-volumes for analysis. In each sub-volume, the algorithm located the apical and basal sides of the epithelium. The epithelium was sub-divided into ten bins along the z-axis relative to its local thickness. Background subtraction was performed on the corresponding bacterial fluorescence trace for each sub-volume. The remaining fluorescence intensity of bacteria, which served as a proxy for the number of bacteria, in each z-bin was integrated. The sum of local profiles across all sub-volumes provided the final fluorescence intensity as a function of traversal distance for a particular field.

**Figure 6:**
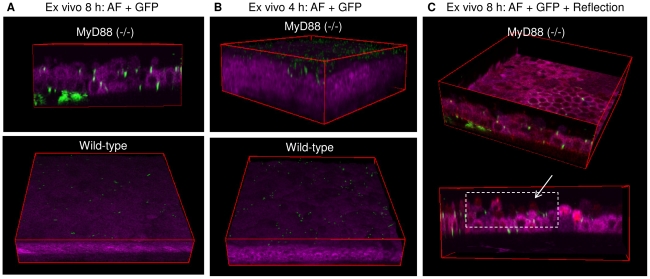
Impact of MyD88 deficiency on defense against epithelial traversal by bacteria after blotting. Panel (**a**) shows two-photon images of cellular NAD(P)H autofluorescence (magenta) of MyD88 (−/−) (**top**) and wild-type (**bottom**) mouse corneas. Deep bacterial traversal (GFP, green) of corneal epithelium was detected in MyD88 (−/−) but not wild-type mice 8 h after bacterial challenge *ex vivo*. Only adherent bacteria without epithelial traversal were found in tissue paper-blotted wild-type corneas. Panel (**b**) shows an earlier (4 h) time-point at which a relatively larger number of bacteria were found adhering to the MyD88 (−/−) corneal epithelium as compared to the wild-type. In panel (**c**), merged image (fuchsia) of reflection (red) and autofluorescence (magenta) is shown. Reflection confocal methodology (red) can be used to capture cells not visualized by autofluorescence (i.e. dying or dead cells) to monitor cell viability during bacterial traversal. This method confirmed that penetrated bacteria at the later (8 h) time point were overlaid with epithelial cells.

Figure 5B shows a quantitative comparison of wild-type to MyD88 knockout mice on traversal after tissue paper blotting to allow adherence (no EGTA treatment). The impact of MyD88 deficiency was found to be similar to EGTA treatment, i.e. it enabled bacteria to traverse deeply through the blotted, multilayered corneal epithelium with a significant number reaching the basal lamina by 8 h post-inoculation as compared to wild-type controls that showed no significant traversal (Figure 6A and Video S7).

Close inspection of the NAD(P)H AF images suggested that the epithelium of MyD88 knockout murine corneas was altered after the blotting and the subsequent 8 h of bacterial challenge. Many of the epithelial cells were rounded, and the total number of cell layers appeared reduced. While cell rounding was also seen after bacteria had traversed EGTA-treated corneas (Figures 2 and 3), the apparent “thinning” of the entire epithelial layer was observed only for challenged MyD88 knockout eyes. To explore the mechanism for these morphological changes, experiments were repeated using a shorter challenge time frame (4 h versus 8 h). The data showed unusually large numbers of bacteria adhering to the surface of MyD88 knockout corneas at 4 h, without significant traversal (Figure 6B and Video S8). MyD88 mutation enhanced bacterial adhesion at time points earlier than it enabled traversal. Further, the underlying corneal epithelium appeared morphologically normal at the earlier (4 h) time point. Thus, changes to epithelial morphology noted at 8 h must have occurred *after* bacteria bound, and were probably caused by the bacteria, rather being a direct result of MyD88 mutation. Indeed, we might expect cells lacking MyD88 (important in innate defense responses) to be more sensitive to negative effects of bacteria than “normal” EGTA exposed cells.

Use of the reflection method in combination with NAD(P)H autofluorescence allowed the impact of 8 h of bacterial challenge on cell viability to be examined (Figure 6C and Video S9). That data showed that the apparent “thinning” of the epithelial cell layer was actually due to loss of metabolic activity of many of the surface cells; i.e. some cells were visible by the reflection method, but not by NAD(P)H AF. While there were some bacteria located at the interface between non-active (visible by reflection) and metabolically active (visible by NAD(P)H autofluorescence), bacteria were also found located between and below metabolically active cells, suggesting that bacterial traversal was not simply a consequence of cell death, despite the noted changes to cell viability.

**Figure 7:**
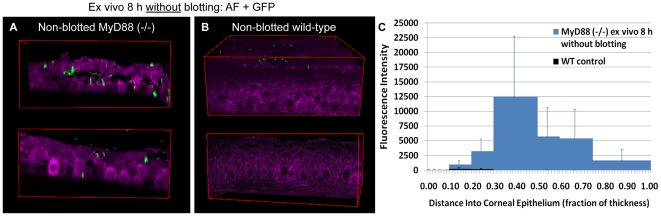
Impact of MyD88 deficiency in the absence of blotting. Panels (**a**) and (**b**) show two-photon images of cellular NAD(P)H autofluorescence (magenta) in non-blotted MyD88 (−/−) or wild-type mouse corneal epithelium 8 h after bacterial challenge *ex vivo*. Significant bacterial adherence and traversal (GFP, green) were found in MyD88 (−/−) mouse (**a**) but not wild-type mouse corneas (**b**). This observation was confirmed by quantitative localization of GFP bacteria using the custom program as shown in panel (**c**).

Since blotted MyD88 knockout corneas showed increased susceptibility to bacterial adhesion and bacterial traversal compared to wild-type, we next explored its impact in the absence of blotting. Surprisingly, the corneal epithelium of freshly excised MyD88 mutant eyeballs was susceptible to both bacterial adhesion (5752 ± 2601 cfu/mm^2^) and subsequent bacterial traversal (Figure 7A, 7C) without the need for blotting, or any other form of manipulation. In contrast, bacteria only occasionally adhered to wild-type control mouse corneas (672 ± 319 cfu/mm^2^) under the same circumstances (Figure 7B, 7C).

**Figure 8:**
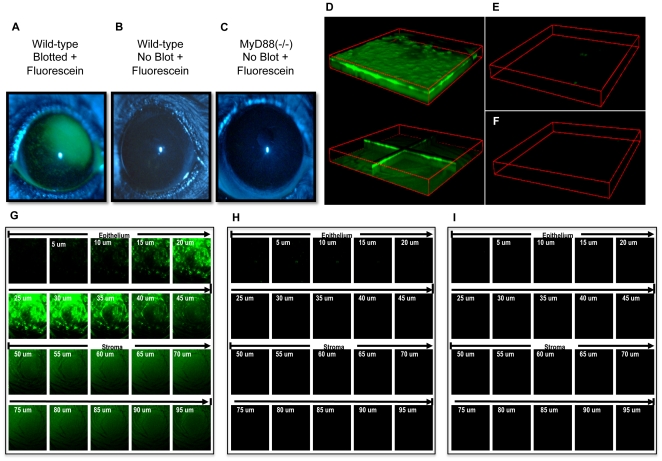
MyD88 corneas do not label with fluorescein suggesting tight junctions are intact *ex vivo* and *in vivo*. Fluorescein was added to tissue paper blotted or normal healthy (non-blotted) mouse corneas to access cell-cell junction integrity. Extent of corneal staining was examined using a slit lamp (**a-c**) and confocal microscopy (**d–i**). Panels (**a, d, g**) show extensive fluorescein staining (green) in blotted wild-type (C57BL/6) corneas, but not non-blotted wild-type (**b, e, h**) or MyD88 (−/−) (**c, f, i**) mouse corneas. Z-stack confocal images (1272 µm × 1272 µm × 100 µm) are presented as 3D block view (**d, top**), 3D orthoslice view (**d, bottom**), and 2D x-y view (**g**) for blotted and fluorescein stained wild-type mouse cornea. In parallel, 3D block views and 2D x-y views of confocal images are shown for normal wild-type (**e, h**) and MyD88 (−/−) (**f, i**) mouse corneas. These data show that epithelial tight junctions are intact in MyD88 knockout corneas prior to bacterial exposure.

A potential mechanism for MyD88 knockout corneal susceptibility to bacterial adhesion and subsequent traversal would be if the lack of MyD88 disrupted epithelial junctional integrity. To explore that possibility, MyD88 knockout and wild-type mice were compared for corneal epithelial susceptibiity to fluorescein staining/penetration, a test used widely in epithelial cell culture, whole tissues, and in the eyes of human patients in routine clinical practice, to assess epithelial permeability/junctional integrity [32]–[34]. Confocal microscopy was used to determine depth of fluorescein penetration. Even without subsequent EGTA treatment, the epithelium of blotted wild-type mouse corneas was susceptible to deep penetration by fluorescein (Fig. 8). In contrast, neither wild-type nor MyD88 knockout corneas labeled with fluorescein when not blotted (Fig. 8). Therefore, susceptibility of unblotted MyD88 knockout corneas to bacterial adherence and traversal was not due to disruption of epithelial tight junctions prior to bacterial exposure. Further, the data suggest that tight junctions do not act in isolation to modulate bacterial traversal in wild-type eyes. Whatever the case, these two models enable traversal by different mechanisms, which will provide additional tools for subequent studies of epithelial defense against infection.

Since bacteria bound to (and penetrated) unblotted MyD88 deficient corneas, MyD88 is required for defending the healthy corneal epithelial surface against bacterial adhesion, in *addition* to protecting it against bacterial traversal. How these findings reconcile with the established role of surface-associated mucins in protecting against bacterial adhesion is not yet clear.

**Figure 9:**
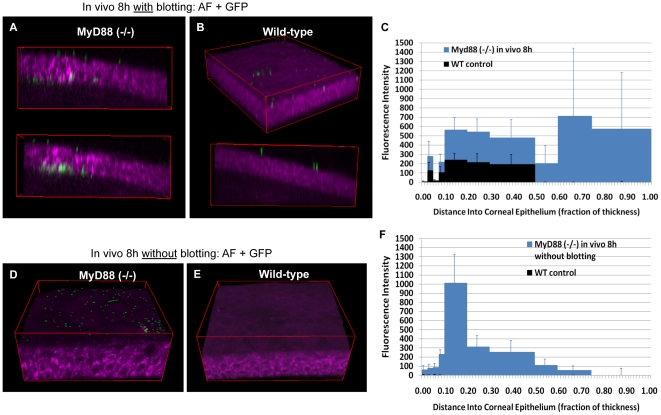
Impact of MyD88 deficiency on colonization *in vivo*. Similar to the *ex vivo* results at the same time-point, panels (**a**) and (**b**) show blotted corneas of MyD88 (−/−) mice were more susceptible to both bacterial adherence and traversal after 8 h of bacterial challenge as compared to blotted wild-type which showed bacterial adherence only. This observation was confirmed by quantitative localization of GFP bacteria using the custom program as shown in panel (**c**). Whereas in the absence of blotting, panels (**d**) and (**e**) show bacterial traversal *in vivo* was disabled. Only bacteria adherence was detected on MyD88 (−/−) (**d**) but not wild-type (**e**) mouse corneas. This observation was confirmed by quantitative localization of GFP bacteria using the custom program as shown in panel (**f**).

The above studies were done using freshly excised eyeballs. We have reported that tear fluid, which bathes the corneal epithelium *in vivo*, is protective against bacteria [35], [36]. In a more recent *in vitro* study we showed that tear fluid can modulate epithelial immunity directly to protect cells against various bacterial virulence strategies, involving upregulation of both RNase7 and ST2 [37]. Thus, we performed experiments in which the blotting and bacterial challenge steps were both performed while the eye was *in vivo*. Experiments were done both with and without blotting using 8 h of bacterial challenge, and both wild-type (control) and MyD88 knockout mice. With blotting, the *in vivo* results mirrored the *ex vivo* results, i.e. both wild-type and MyD88 knockout mouse corneas bound bacteria, with more extensive binding and also epithelial traversal by bacteria for the MyD88 mutant eyes (Figure 9A–C). As expected, bacteria did not associate at all with non-blotted wild-type corneas *in vivo* (Figure 9E, 9F). MyD88-deficient corneas showed only low level bacterial adhesion and only shallow bacterial penetration beyond the surface (Figure 9D, 9F). This contrasted with results obtained for experiments done *ex vivo* that yielded significant adhesion and penetration to the basal lamina. Indeed, *ex vivo* data showed maximal GFP fluorescence intensity was 12,000 at a depth of 0.4 towards the basal lamina (Figure 7); *in vivo* it peaked at only 1,200 and at a more shallow level of 0.2 (Figure 9F). Thus, the unmanipulated MyD88-deficient corneas were less susceptible to bacterial colonization *in vivo*, than they were when removed and challenged *in vitro*. Whether this is explained by (MyD88-independent) protective biochemical factors *in vivo*, or simply by physical removal of bacteria during blinking/tear flow, is yet to be established.

### Blotting/EGTA pretreatment versus MyD88 knockout mice as models for enabling traversal to be studied

While useful for studying defenses against traversal, these models also provide systems that could be used to enable the traversal process to be studied (e.g. identifying bacterial factors involved). The two approaches have different advantages; i.e. blotting/EGTA treatment can be used with mice of any background (i.e. wild-type, gene knockouts, or transgenic mice such as the CFP membrane mice), while use of MyD88 knockouts does not necessitate chemical or other treatment of the tissue to enable traveral.

Given the known functions of MyD88, its role in defense against both bacterial adhesion and bacterial traversal are likely to involve either TLR- or IL-1-mediated epithelial-, or resident macrophage or dendritic cell-, derived/initiated innate defenses. These could include antimicrobial peptides, or infiltrating phagocytes (*in vivo*) [38]–[43]. Junctional integrity has also been shown to be responsive to TLR-, and MyD88-, mediated signaling in intestinal epithelia [44]. While the data in Fig. 9 show normal barrier function in naïve MyD88 knockout mice (and therefore that tight functions are functionally intact), it remains possible that regulation of junctional integrity in the face of bacterial challenge is compromised in the corneas of MyD88 knockout animals. Other possibilities are that mucins (or other epithelial surface-associated moieties that either bind or repel bacteria) are dependent on MyD88, or that they act in synergy with MyD88-mediated factors (such as antimicrobials) to exert their protective effects. Further research will be needed to sort through these and other potential possibilities for MyD88 involvement in epithelial defense against traversal by microbes.

### Conclusion

In this report, we demonstrate that the combination of various imaging methods can be used to further our understanding of bacterial-host cell interactions in live unprocessed tissues. These relatively simple and robust non-invasive methods, used with strategies for manipulating tissue susceptibility to microbes, and a custom data analysis program, represent a set of novel, quantitative tools for studying both pathogenesis of, and defenses against, infection. Using them, we have shown directly that MyD88 plays a critical role in defense against epithelial colonization by a Gram-negative opportunistic pathogen, and demonstrating that host-pathogen interactions reported to occur *in vitro* (e.g. membrane bleb-niche formation) can also occur when tissues are challenged intravitally. The data presented also suggest new insights into bacterial pathogenesis not previously reported (such as a spatio-temporal relationship between bacteria and collagen fibrils during infection), that can now be expanded upon using the developed methods. While there are clear advantages to using mice, including the availability of gene knockouts and other reagents, these methods could be used with eyes from other species or with other types of tissue. With the correct combination of resources, and if image stabilization can be achieved at the high magnification needed to detect infecting bacteria, these methods could be implemented *in vivo* using anesthetized live animals as has been done for studying immune cell trafficking [45]. Since these imaging methods are non-invasive, there could even be potential for translation to use in humans.

## Materials and Methods

### Ethics Statement

All procedures involving animals were performed in strict accordance with a protocol approved by the Animal Care and Use Committee, University of California, Berkeley, which is an AAALAC accredited institution. Protocol #R203-1210B.

### Source of Mice

MyD88 (−/−) mice fully backcrossed to a C57BL/6 background were provided by Dr. Greg Barton (University of California, Berkeley, CA). C57BL/6 transgenic mice expressing membrane-bound CFP under the control of human ubiquitin C promoter were also used in some experiments [46]. Age-matched C57BL/6 and Black Swiss wild-type mice were purchased from Taconic Farm Inc. (Hudson, NY).

### Bacterial strain and inoculum preparation

*Pseudomonas aeruginosa* invasive strain PAO1 expressing enhanced GFP (PAO1-GFP) from a highly stable, multicopy plasmid (pSMC2) was used [47] (kindly provided by Dr. Gerald B. Pier, Harvard Medical School). Plasmid transformation of bacteria was achieved by standard electroporation. Bacteria were grown on tryptic soy agar plates supplemented with carbenicillin 300 µg/ml at 37°C for ∼16 h. Inocula were prepared by suspending bacteria in Dulbecco's Modified Eagle's Medium (Lonza, Walkersville, MD) to a concentration of ∼10^8^ or 10^11^ cfu/ml. We have previously confirmed that the bacteria can retain this, and other, plasmids *in vivo* even without antibiotic selection (up to 48 h and longer) [48], [49], and show strong fluorescence using conventional and confocal fluorescence microscopy.

### *Ex vivo* traversal model

C57BL/6 wild-type and MyD88 (−/−) gene knockout mice were subject to lethal injection with anesthesia cocktail (21 mg/ml ketamine, 2.4 mg/ml xylazine, and 0.3 mg/ml acepromazine). Whole eyes were enucleated to exclude tear fluid before a PBS rinse (∼10 ml), tissue paper blotting (when used) with Kimwipe™ (Kimberly-Clark) and incubation with 200 µL of bacterial suspension (∼10^11^ cfu/ml) at 35°C for 4 or 8 h before imaging. In some experiments, blotted eyeballs from transgenic CFP-membrane mice and Black Swiss wild-type mice were treated with EGTA (100 mM in PBS) or PBS alone for 1 h before bacterial incubation for 6 h (both treatments at 35°C).

### *In vivo* traversal model

C57BL/6 wild-type and MyD88 (−/−) gene knockout mice were subject to anesthesia by intraperitoneal injection (50 µl/25 g of body weight) of ketamine/xylazine/acepromazine cocktail. One cornea of each of the anesthetized mice were rinsed with PBS to wash away tear fluid, then blotted with Kimwipe™ tissue paper (when used) and inoculated with 5 µl of bacterial suspension (∼10^11^ cfu/ml). Animals were euthanized 8 h post-inoculation by lethal injection, and eyes were carefully enucleated, then rinsed with PBS (∼10 ml) prior to imaging.

### Scratch infection model

One cornea of anesthetized mice was scratched linearly three times using a sterile 25-gauge needle, then inoculated with 5 µl of bacterial suspension (∼10^8^ cfu/ml). Animals were euthanized 24 h post-inoculation when corneal opacity had become obvious. Eyes were carefully enucleated and rinsed with PBS prior to imaging.

### Technologies for visualization of bacteria and host cells

GFP-expressing bacteria were excited by the 488 nm argon ion laser line (different fluorophores, e.g. RFP, can be used for bacteria as needed) or the multiphoton laser at 920 nm (in opaque mouse corneas). To visualize corneal epithelial cells in live unprocessed eyeballs, a combination of previously described two-photon and confocal imaging techniques were used [50], [51]: (1) Multiphoton excitation at 720 nm from the Ti:Sa laser with a pulse width of ∼100 fs and maximum average power of ∼1.6 W into the microscope, which excites endogenous NAD(P)H molecules in metabolically active living cells [17]–[19]. Corneas were previously used to show the potential of *in situ* NAD(P)H autofluroescence (AF) *via* two-photon microscopy for assessing cellular metabolic states [17]. (2) HeNe laser line at 633 nm, which allows imaging of both live and dead cells by detecting reflected light [51]. (3) Argon ion laser line at 458 nm, which excites membrane-bound CFP expressed in transgenic mice.

### Combined two-photon/confocal imaging of whole eyeballs

Mouse eyeballs examined for bacterial traversal were rinsed with PBS three times to remove non-adherent bacteria. After rinsing, the back of an eyeball (where the optic nerve is located) was fixed on a 12 mm round glass coverslip with cyanoacrylate glue to maintain an upright position (i.e. cornea facing up). The coverslip/eyeball was placed in a 47 mm Petri dish which was then filled with Ham's F-12 medium (Lonza, Walkersville, MD) to cover the eyeball completely, and was ready for imaging using a 63x/0.95 NA water-dipping objective of an upright two-photon/confocal microscope (LSM 510 META NLO Axio Imager; Carl Zeiss, Thornwood, NY) equipped with a tunable, ultrafast and dispersion-compensated Ti:Sapphire laser (Mai Tai DeepSee; Spectra-Physics, Santa Clara, CA). A 435–485 nm bandpass or a 500–550 nm bandpass filter was used to detect NAD(P)H AF or GFP respectively. Three or more random fields of each eye were imaged from the corneal surface through the entire epithelium in 0.5 µm steps. Three dimensional images were reconstructed from z-stacks using ImageJ (NIH).

### Computer program for quantitative localization of bacteria

Data analysis was performed with IDL (ITT Corp., Boulder, CO; code available upon request). The epithelium was divided by binning adjacent x-y pixels into typically 1.48 µm x 23.75 µm. Sub-division of the image stack and analysis of the fluorescence profiles as a function of z to locate the apical and basal sides of the epithelium was found to be superior to Sobel and Roberts edge enhancement filters which did not give satisfactory results.

For each sub-volume, we had two relevant channels: one containing the structural information regarding the epithelia (AF, reflection, or CFP), and the other containing bacterial fluorescence (e.g. GFP). AF and CFP give a similar profile, characterized by a single central peak in fluorescence as a function of z, while reflection requires a slightly different strategy to locate the apical/basal surfaces. After sub-division, the AF or CFP data stack was optionally smoothed, depending on the signal-to-noise ratio, using a boxcar average. The central peak was then located using a peak-finding algorithm which computes the derivative of the fluorescence trace by convolution with a Savitsky-Golay filter. The order and number of points of the filter were selected by the user. The peak was located by searching for zero crossings in the derivative. Once located, this point marked the center of the epithelium. At this point, the apical and basal edges of the epithelium were located by using the same peak-finding algorithm to locate the peak values of the derivative of the AF or CFP profile from the beginning of the stack to the center of the epithelium (apical) and from the center of the epithelium to the end of the stack (basal).

When using reflection to locate the surfaces the situation is slightly different. Reflection profiles have two strong peaks: one at the surface of the cornea and one at the basal lamina. Hence, the peak finding algorithm was used to locate these two peaks. The edges of the epithelium were located by peak-searching the derivative of the reflection signal from the beginning of the stack to the surface peak (apical) and from the basal lamina peak to the end of the stack (basal).

To quantify bacterial traversal in each sub-volume, a threshold was used first to determine if bacteria were present. This threshold is set by the user and must be adjusted depending on the particular image stack. If there was significant bacterial fluorescence, the corneal thickness was binned into ten z-bins and the fluorescence is integrated over each bin. This gave a local profile of the bacterial traversal of the epithelium. The sum of the individual profiles gave an overall representation of the traversal in the image stack.

## Supporting Information

Video S1 CFP-membrane mouse corneas were tissue paper blotted, PBS treated for 1 h, then bacteria- challenged for 6 h *ex vivo* before confocal imaging. Moving XZ and XY planes of the 3D image show individual corneal epithelial cells (CFP membrane, magenta) with GFP bacteria (green) adhering on the epithelial surface.
(MOV)

Video S2 Confocal imaging of *ex vivo* blotted, EGTA treated (1 h) and bacteria-challenged (6 h) CFP-membrane mouse corneas. Moving XZ and XY planes of the 3D image show corneal epithelial cells (CFP membrane, magenta) with GFP bacteria (green) deeply traversing the corneal epithelium to the level of the underlying basal lamina.
(MOV)

Video S3 Two-photon and confocal imaging of *ex vivo* blotted, EGTA treated (1 h) and bacteria- challenged (6 h) wild-type Black Swiss mouse corneas. Moving XZ, YZ and XY planes of the 3D image show corneal epithelial cells (cytoplasmic autofluorescence, magenta) with GFP bacteria (green) deeply traversing the corneal epithelium to the level of the underlying basal lamina.
(MOV)

Video S4 3D block view of the confocal image of *ex vivo* blotted, PBS treated (1 h) and bacteria- challenged (6 h) CFP membrane mouse corneas. GFP bacteria (green) were found to adhere to the corneal surface without traversing the epithelium. CFP cell membranes are shown in red.
(MOV)

Video S5 Combined two-photon and confocal time-lapse imaging of GFP bacteria (green) confined in a CFP membrane bleb (cyan) of the corneal epithelium (cytoplasmic autofluorescence, magenta) of transgenic CFP-membrane mouse corneas blotted, EGTA treated (1 h) and bacteria-challenged (6 h) *ex vivo*.
(MOV)

Video S6 Two-photon imaging of cellular autofluorescence (magenta) and GFP bacteria (green) within an opaque infected wild-type mouse cornea. Bacteria are visible in both the epithelium and in the stroma, but the colonized areas are spatially separated by the intervening basal lamina. Those in the epithelium form a circular pattern around cells/cell nuclei, while those in the stroma are seen lined up (single file) along the collagen fibrils.
(MOV)

Video S7 *Ex vivo* blotted and bacteria-challenged MyD88 (−/−) mouse corneas at 8 h time-point. 3D image of the cornea showed GFP bacteria (green) traversed the entire thickness of the corneal epithelium (cytoplasmic autofluorescence, magenta). Traversal was accompanied by disruption of epithelial morphology, but bacteria did not cross the underlying intact basal lamina into the stroma.
(MOV)

Video S8 *Ex vivo* blotted and bacterially challenged MyD88 (−/−) mouse corneas at 4 h time-point. Traversal was not enabled at this shorter time-point, and the epithelium demonstrated normal morphology. However, the MyD88 (−/−) mouse corneas were found significantly more susceptible to bacterial adherence than wild-type at this early time-point.
(MOV)

Video S9 The reflection method was used to enable visualization of both live and dead cells (red), while the autofluorescence method showed only live cells (detects cytoplasmic NAD(P)H of actively metabolizing cells) (magenta). The two channels were superimposed (fuchsia) to confirm that penetrated bacteria (green) in tissue-paper blotted MyD88 (−/−) mouse corneas were actually overlaid with cells, and that some overlying cells remained viable. Note the large number of bacteria at the interface between the epithelium and the stroma (appears red) at the level of the basal lamina barrier.
(MOV)
